# 7th International Conference on Duckweed Research and Applications: Depicting an Era of Advancing Research Translation Toward Practical Applications

**DOI:** 10.3390/plants14142143

**Published:** 2025-07-11

**Authors:** Klaus J. Appenroth, Viktor Oláh, Hidehiro Ishizawa, K. Sowjanya Sree

**Affiliations:** 1 Matthias Schleiden Institute—Plant Physiology, Friedrich Schiller University, 07743 Jena, Germany; 2 Department of Botany, Institute of Biology and Ecology, Faculty of Science and Technology, University of Debrecen, H-4032 Debrecen, Hungary; olahviktor@unideb.hu; 3 Department of Applied Chemistry, Graduate School of Engineering, University of Hyogo, Himeji 671-2280, Japan; ishizawa@eng.u-hyogo.ac.jp; 4 School of Biotechnology, Institute of Science, Banaras Hindu University, Varanasi 221005, India

**Keywords:** duckweed, evolution, genomics, lemnaceae, metabolome, microbiome, practical applications

## Abstract

Duckweeds are aquatic monocotyledonous plants known to be the smallest and the fastest growing angiosperms. The 7th International Conference on Duckweed Research and Applications (7th ICDRA) was held in Bangkok, Thailand, from 12th to 16th November 2024. The conference drew young and experienced scientists from across the world who presented their research in varied fields. This conference report presents the highlights of the advancements in the field of duckweed research and application in the sections: Genomics and Cell Biology; Diversity, Ecology, Evolution; Physiology, Reproduction, Metabolomics; Microbiome and Interactions; Applications; and Future Outlook. The next conference, 8th ICDRA, will be held in Naples, Italy, in 2026.

## 1. Introduction

Duckweeds are the smallest and the fastest-growing angiosperms occupying the lentic ecosystems. These plants have re-emerged as model organisms for research in various fields of plant biology. Duckweeds also showcase immense potential in their practical applicability, owing to their role in human food and animal feed, wastewater treatment, bioenergy production, and so on. Progress made in research and applications of duckweeds in the last two years since the last meeting in 2022 in Gatersleben, Germany [1], was highlighted to the scientific community through numerous presentations (talks, oral (34 in number), and poster (64 in number) presentations). This 7th biennial conference on Duckweed Research and Applications held in Bangkok, Thailand, from 12th to 16th November 2024 was chaired by Arinthip Thamchaipenet, Kasetsart University, and Metha Meetam, Mahidol University, Thailand. There was an increase in both the number of participants as well as the diversity of the topics presented and discussed during the conference. The abstract book of the conference is available online: www.7icdra2024.com/?page_id=2452 (accessed on 6 July 2025).

**Figure 1:**
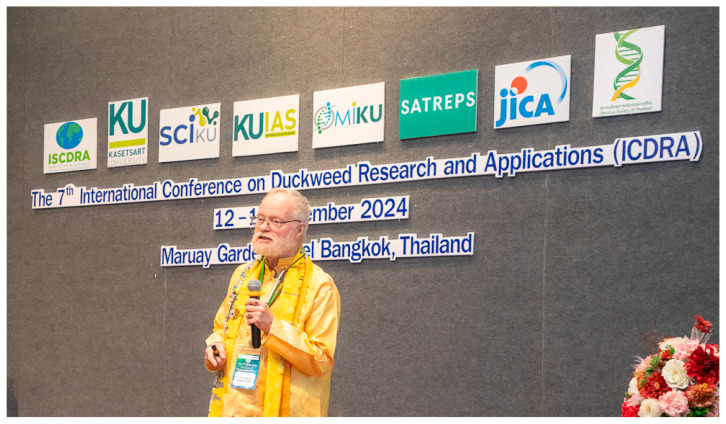
Klaus-J. Appenroth presenting the plenary talk at the 7th ICDRA (Photo courtesy: Conference organizers).

The conference was inaugurated with a plenary talk by one of the authors of this report, Klaus-J. Appenroth, who pointed out several open questions highlighting the bottle necks in the field of duckweed research and applications, which have been presented in “Future Outlook” of this report (Figure 1). The various talks and presentations have been summarized portraying the advancements in each area under the following chapters in this report: Genomics and Cell Biology; Diversity, Ecology, Evolution; Physiology, Reproduction, Metabolomics; Microbiome and Interactions; Applications; and Future Outlook. Moreover, practical demonstrations and tours to the several facilities showcasing the applications of duckweeds in Thailand was highly appreciated.

**Figure 2:**
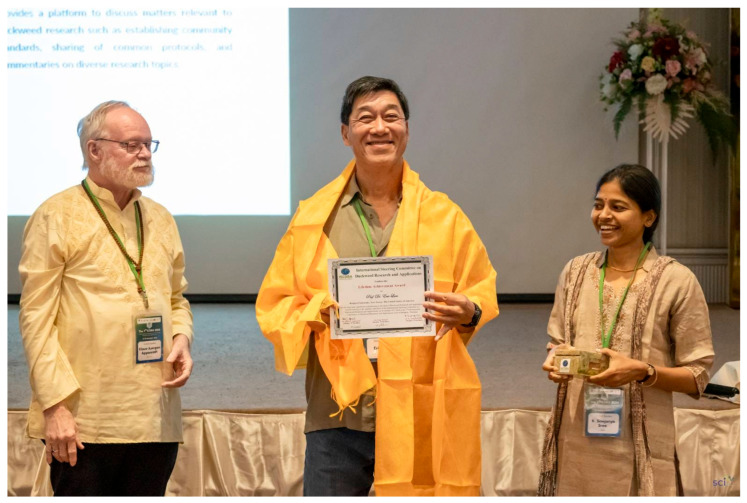
Eric Lam (center) receiving the Lifetime Achievement Award conferred by the ISCDRA (Left: Klaus-J. Appenroth; Right: K. Sowjanya Sree; Photo courtesy: Conference organizers).

Apart from the scientific sessions, during the meeting, the International Steering Committee on Duckweed Research and Applications (ISCDRA) conferred the Lifetime Achievement Award to Prof. Dr. Eric Lam, Rutgers University, New Jersey, USA, for his long-term significant contributions to the field of Duckweed Research and Applications and for serving as the member and Chair (three terms) of the International Steering Committee on Duckweed Research and Applications (Figure 2).

**Figure 3:**
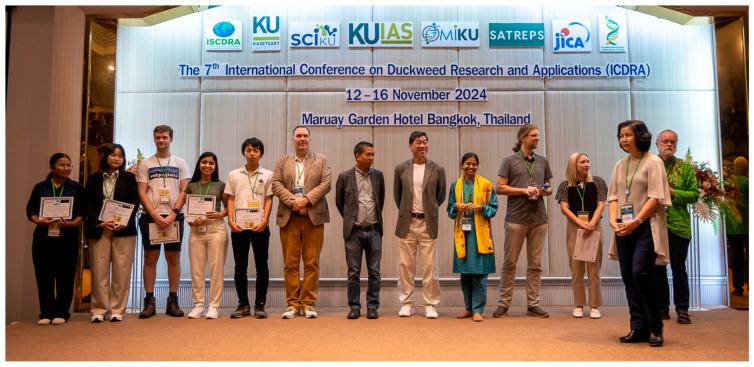
Student awardees together with the Jury, Sponsors, and conference organizers (Photo courtesy: Conference organizers).

The student *presentations*, both oral ones and posters, were encouraged by presenting awards (Figure 3). A Best Oral Presentation Award was presented to Ms. Kari Lagan from The Pennsylvania State University, USA, for her talk entitled “*Leveraging acidification pretreatment of dairy digestate to increase duckweed protein yields while decreasing ammonia volatilization and water use*” sponsored by the MDPI journal *Plants*. Five poster presentation awards, sponsored by *Sustainable Planet*, were presented to Ewout Crombez, Ghent University, Belgium (“*The impact of polyploidy on intra-specific genome variation in Lemna aequinoctialis*”); Sara Nouere, University of Mainz, Germany (“*Real-time evolution of plant chemical defenses in natural communities*”); Riku Takei, Hokkaido University, Japan (“*Isolation and application of plant growth promoting bacteria for production using methane fermentation digested liquid*”); Sunisa Srisopa, Khon Kaen University, Thailand (“*Biogas production performance on co-digestion of tapioca starch factory wastewater and duckweed by continuous stirred tank reactor*”); and Hathaipat Thongthung, Kasetsart University, Thailand (“*Use of duckweed as a feed ingredient*”).

The 6th ISCDRA was constituted during this meeting, with the newly elected members: Eric Lam, Rutgers University, USA; Marcel Jansen, University College Cork, Ireland; Klaus-J. Appenroth, Friedrich Schiller University Jena, Germany; Asaph Aharoni, Weizmann Institute of Science, Israel; Tsipi Shoham, Greenonyx, Israel; and K. Sowjanya Sree, Banaras Hindu University, India. K. Sowjanya Sree was elected as the Chair of the 6th ISCDRA.

In the following, recent advancements in various fields of duckweed research and applications presented as invited talks and oral presentations during the 7th ICDRA have been reported. The next conference, the 8th ICDRA, will be held in Naples, Italy, in 2026.

## 2. Genomics and Cell Biology

The first presentation in this first session was based on the recently available genomic data of a large number of species from all five genera of Lemnaceae (summary in [2].) Todd P. Michael, Salk Institute for Biological Studies, La Jolla, CA, USA, considered the morphological reduction from *Spirodela* to *Wolffia* during evolution from a genomic point of view (“*Duckweed super-pangenome*”). The available genomes made it possible to map architectural genome changes to the changes in morphological features. The creation of a duckweed “super-pangenome” allows to trace the loss and gain of whole gene families connected with these morphological developments. The dream is to model a virtual, in silico plant, which can be used for predicting potential applications. Robert A. Martienssen, Cold Spring Harbor Laboratory NY, USA, reported genome evolution from *Spirodela* to *Wolffia* by comparison with other aquatic and terrestrial plants, discovering features, e.g., frequent hybrid polyploidy, lack of stomatal closure at high CO_2_ levels or accumulation of oxalate (“*The genomes and epigenomes of aquatic plants (Lemnaceae) promote triploid hybridization and clonal reproduction*”). Some genes involved in RNA interference that are required for the so-called triploid block explain the frequent existence of triploid *Lemna* clones [3]. The high growth rates of several Lemnaceae clones make them good candidates for powerful biotechnological platforms, e.g., for the production of high oil-containing *Lemna*. For the first time, single-cell transcriptomics was performed in duckweeds using *Wolffia australiana*, which is probably one of the most structurally reduced flowering plant [4]. Tom Denyer, University of Tuebingen, Germany, discovered that cells of this species could be divided on this experimental basis into four principal types, parenchyma and epidermis, above and under water, although some minor specializations are seen within these groups. During two points of time-of-day transitions the core circadian clock machinery was investigated (“*Streamlined spatial and environmental expression signatures characterize the minimalist Wolffia australiana*”). This method has a huge potential to investigate developmental processes. This was exemplified by Shuqing Xu, University of Mainz, Germany, using *Wolffia microscopica* (“*Single nuclei sequencing of Wolffia microscopica reveals the minimal control of flower development in plants*”). What they called as “single-nuclei multiome” based on RNA-seq and ATAC-seq (“*Assay of Transposase Accessible Chromatin sequencing*”) of flowering fronds of *W. microscopica* [5] resulted in 16 nuclei clusters, containing, e.g., transcription factor genes for pollen cell-type. Other cell types related to sexual reproduction will be investigated in the future. Alexander Ware, University of Nottingham, UK, is also interested in the morphological structure of duckweeds, especially in the composite body. He followed in Late E. Landolt’s footsteps who described the fronds of duckweeds “as a composite structure containing a compressed shoot from the stipe-frond junction to the node, with the distal section representing a leaf-like structure” [6]. He investigated transcriptomics signature of these morphological structures of *Spirodela polyrhiza* and *Lemna minor* and compared them with those of the plants with more clear structural plans like *Pistia stratiotes* and *Colocasia esculentum* (“*Resolving the duckweed frond with comparative transcriptomics*”). As a result, he could confirm the morphological interpretation of Lemnaceae fronds by Late E. Landolt [6] using transcriptomic data.

## 3. Diversity, Ecology, Evolution

The first invited talk of the section, Robert A. Laird from University of Lethbridge, Canada, summarized their latest results on duckweed senescence (“*Duckweeds as a model system for plant senescence*”). For more than seven decades, duckweeds have been models in plant aging studies [7], and the new findings demonstrated that parental age did not only affect the offspring’s lifespan (Lansing effect), but also had a negative impact on total reproduction, projected growth rate, and realized population growth. Besides parental age, caloric intake could also influence the individual lifespan of animal organisms. The recent results by the Laird group revealed an even broader relevance of this rule, indicating that caloric restriction could extend the lifespan of duckweed fronds too. Moreover, they found highly similar patterns in lifespan distributions of short- and long-lived plants (temporal scaling) that was applicable to both caloric restriction and temperature treatments.

The second invited talk, entitled “*Keep it simple Spirodela: structural reduction in duckweed*”, by Anthony Bishopp, University of Nottingham, UK, depicted duckweed evolution as a progressive loss of vestigial features that were otherwise common amongst flowering plants. Duckweeds had gone through a dramatic evolutionary reduction during their adaptation to aquatic habitats [8], and this trend was presented through the gradual simplification and eventual loss of roots in parallel with a decreasing number of auxin receptors and responsivity to auxin. Similarly, Yanling Jin, Chengdu Institute of Biology, China, described duckweed evolution as a reverse trajectory of terrestrial plants conquering freshwaters again (“*Duckweed evolution: from land back to water*”). Besides simplification of root structure and decreasing gene numbers of the main phytohormonal pathways, further traits were also evidenced in the multiproxy study to support aquatic re-adaptation, such as the gradually decreasing lignocellulose content, the changing distribution and development of stomata, and the enhanced flavonoid biosynthetic pathway.

Besides the major evolutionary trends, family affairs of Lemnaceae have also been puzzling scientists for generations [9]. The reduced morphology, wide phenotypic plasticity, polyploidy, and recently recognized interspecific hybrids make it challenging to reveal the actual phylogenetic relationships of different accessions, which would be one of the first necessary steps in valorizing large-scale duckweed germplasm collections. Laura Morello from the Institute of Agricultural Biology and Biotechnology, Italy, evidenced through the example of their CNR-IBBA duckweed stock collection in Milan, Italy, that joint analyses of nuclear and plastidic genetic markers combined with genome size estimation may help in disentangling the complexity of different duckweed genera. In her talk (“*So small, so similar, so different: investigating genetic diversity in duckweeds*”), she also proposed an introduction of alternative taxonomic ranks (e.g., species complexes) to better describe interspecific gene flow through hybridization, and she raised the possibility that distantly related single accessions in duckweed collections may represent so far undescribed species. Similarly, Ingo Schubert from the Leibniz Institute of Plant Genetics and Crop Plant Research in Germany pointed out the necessity of deciphering phylogenetic relationships within the *Lemna*, *Wolffia*, and *Wolffiella* genera. As he presented in his talk (“*Hidden promiscuity explains duckweed diversity and evolution*”), besides further facilitating commercial applications of duckweeds, multi-approach phylogenetic studies can also help in addressing such basic questions as whether triploid and dihaploid hybrid lineages are evolutionary dead ends or can still return to sexuality. Athita Senayai, Kasetsart University, Thailand, focused on the local duckweed flora of Thailand (“*Genetic and morphological variation among populations of duckweed species from Thailand*”). By analyzing a total of 38 duckweed samples from 26 locations, they identified four species (*S. polyrhiza*, *Landoltia punctata*, *Lemna aequinoctialis*, *Wolffia globosa*), each representing a different genus. Besides obtaining an insight into the genetic diversity of those four species in Thailand, they could also link the morphological traits to genetic variations that may support clone selection in future duckweed research and applications.

Duckweeds have long been known to display large intraspecific variation in ploidy levels [10], thus providing an ideal platform to study the effects of Whole Genome Duplication (WGD). Quinten Bafort, Ghent University, Belgium, summarized their latest results with neopolyploid *S. polyrhiza* lines (“*Experiments with neopolyploids unveil the ecological and evolutionary significance of polyploidy in duckweeds*”), explaining the importance of this macromutation on phenotype and fitness. In their common garden experiments, the artificially induced tetraploid duckweed lines often showed strain-specificity in metabolome, stress tolerance, trait plasticity, growth rates, or competitive abilities, but the evidence pointed to the establishment of more tolerant variants in the longer run as a result of recurring polyploidization events in populations under stress. Duckweeds can also help as models in better understanding the evolution, organization, and regulation of the plant genome [1]. As Nikolai Borisjuk from the Institute of Cell Biology and Genetic Engineering, Ukraine, presented in his talk (“*Molecular architecture of 5S ribosomal DNA loci in Spirodela polyrizha: novel insights into plant rDNA evolution and regulation*”), they could successfully describe nucleotide-level architecture of the ribosomal DNA (rDNA) for the first time in a higher plant. Their results with *S. polyrhiza* revealed two different 5S rDNA loci in the genome of this species, with different copy numbers and putatively locus-specific regulation. These findings will hopefully pave the way further to keep duckweeds as fundamental experimental platforms for plant biology research in the post-genomics era [11].

## 4. Physiology, Reproduction, Metabolomics

In the first report in this session, Shu-Nong Bai, Peking University, China, presented an alternative concept to describe the morphology of duckweed (“*Nomenclature, its implication and interpretation of reality—Wolffia demonstrates that a plant is a colony*”; [12]). He suggested replacing the terms like “colony” with “developmental unit”, e.g., in *Wolffia* “generations” (mother and daughter) with “branches”. K. Sowjanya Sree, Banaras Hindu University, India (“*Polyploids of Lemna gibba*”), described a spontaneous, tetraploid mutant of *Lemna gibba* originating from the wild type G3 (7796) in the lab of the Late Jitendra P. Khurana (University of Delhi, India). The mutant showed enlarged sizes of different cell types and differed in stress responses like flowering under different light conditions [13]. Also, in some *L. gibba* clones, different light intensities influenced not only the response at the population level but especially showed pattern-specificity over the surface area of mother and daughter fronds, as presented by Viktor Olah, University of Debrecen, Hungary (“*Frond-level patterns and clonal differences in the light acclimation of Lemna gibba*”; cf. [14]). Progress in cryopreservation was reported by Anton Peterson (IPK, Gatersleben, Germany: “*Viability of duckweed fronds after cryopreservation depends on developmental stages of plastids*”; [15]). He showed that only frond meristems, which comprise pro-plastids, tolerate cryopreservation and provide regrowth after rewarming, while the rest of the parts of cryopreserved duckweed fronds die. *Lemna gibba* containing a luciferase gene was used to investigate circadian regulation. Bioluminescence rhythm was presumably caused by coupled uptake of luciferin from the culture medium (Tokitaka Oyama, Kyoto University, Japan: “*Circadian regulation in the association of physiology of duckweed and its aquatic environment*”). *Wolffia microscopica* shows a short lifespan on media containing ≥0.5 mM ammonium. Therefore, this species was used to screen for anti-aging microorganisms. The group of Jiaming Zhang (Chinese Academy of Tropical Agricultural Sciences, Haikou, China) isolated a strain of *Aspergillus sclerotiorum* ITBB2-31 from rubber tree and applied filter-sterilized or autoclaved exudates on *W. microscopica*, which significantly increased the lifespan. Using the overlapping and effective metabolites from filter-sterilized and autoclaved exudates, the number of possible chemical compounds could be restricted (“*Screening method and metabolic analysis of plant antiaging microorganisms* via *ammonia-induced senescence in the duckweed Wolffia microscopica*”). Innovations in re-circulating indoor vertical farms were reported by Finn Petersen (University of Applied Sciences, Osnabrueck, Germany). A newly developed controller improved uniformity in growth parameters and single nutrient controller and dosing systems addressed the problem of nutrient imbalance, which arises when only electrical conductivity and pH are used as controlling signals [16] (“*Advancing duckweed production: Technical and nutrient management innovations in re-circulating indoor vertical farms*”). Gadolinium is a rare earth element of the lanthanide series. It is very rare in nature but its increasing use, e.g., as magnetic contrast element in magnetic resonance imaging (MRI) and magnetic resonance angiography (MRA) results in environmental contamination. Sandor Szabo (University of Nyiregyhaza, Hungary; [17]) reported the use of *L. gibba* for detoxification of waste (“*Biofiltration, recovery and toxicity of chelated and ionic gadolinium forms using duckweed Lemna gibba*”). For gadolinium-based contrast agents (GBCA) that contain Gd in chelated, non-toxic form, concentration-dependent infiltration was detected but not bioaccumulation. For ionic Gd^3+^, the inhibition of growth rate was at an E_i_C_50_ of 12.4 mg L^−1^. The bio-concentration factor was up to 1100. The authors concluded that in constructed wetlands, GBCA cannot be removed effectively by *L. gibba* but for free Ga^3+^, bioremediation is possible. The largest number of duckweed-related publications are always about phytotoxicity, very often using inhibition of growth as the most important parameter. Paul Ziegler, University of Bayreuth, Germany, asks in his report which test methods have the potential to provide information about the toxic substance present in the aquatic environment (“*Progress in toxicity testing with duckweeds*”). He concludes that several newly applied techniques like fluorescence measurements or the root-regrowth test provide essential advantages concerning time or labor input [18] but the highest potential to acquire toxicant-related information are transcriptomic-, proteomic-, or metabolomic-based and their combinations—resulting in high costs.

Bioregenerative life support systems (BLSS) are becoming a necessity for long-term food production directly in space and duckweeds provide excellent candidates for BLSS application. One basic problem is the response of plants to altered gravities. Leone E. Romano, University of Naples, Italy, investigated the effects of microgravity, hypergravity and simulated Moon gravity on clones of *W. globosa* and *W. australiana* [19]. Some clones of *W. globosa* show only minimal responses of growth, where growth patterns can be analyzed using machine learning techniques [20], and nutrient composition toward changes in gravity. This resilience makes *W. globosa* a strong candidate for bioregenerative life support systems (“*Unraveling Wolffia’s potential for manned interplanetary space missions: growth and genetic responses to altered gravity*”).

## 5. Microbiome and Interactions

One emerging research area in duckweed–microbe interactions is the use of synthetic community (SynCom), a method that provides a controlled way to study microbiome complexity. In the first invited talk, Eric Lam (Rutgers University, USA) introduced this reductionist approach and described the development of a duckweed-based SynCom, composed of 30 distinct bacterial strains coexisting on sterilized duckweed (“*DABs and SynComs—from unraveling microbial ecology to designing ducked holobionts*”). The key advantage of SynCom is their greater controllability compared to natural microbiomes. By systematically analyzing the metabolic capabilities of bacterial strains based on genome sequences, it is becoming possible to predict intricate microbial interactions, such as competitive exclusion mechanisms. This approach enables more precise identification of critical compounds for effective duckweed microbiome management. The next invited talk by Hidehiro Ishizawa, University of Hyogo, Japan, reported findings on the community assembly mechanisms of a six- or seven-member SynCom that maintains stable community structure on *Lemna japonica* (“*Community assembly in synthetic duckweed microbiome*”). This SynCom was strategically designed to mimic the family-level structure of natural duckweed microbiomes, with each bacterial strain representing dominant bacterial families found in environmental samples [21]. Leveraging the simplicity and controllability of the SynCom, their research quantitatively evaluated microbial interspecies interactions in all possible combinations [22]. Through the analysis of this dataset, they concluded that community assembly in duckweed microbiome could be predictively modeled from lower-level information such as interspecies interactions.

Another critical step in advancing our understanding of duckweed–microbe interactions is the development of molecular tools to study microbiome diversity [23]. In the invited talk by Arinthip Thamchaipenet, Kasetsart University, Thailand, the use of full-length 16S rDNA sequencing and metagenomic sequencing to analyze real duckweed microbiome was reported (“*Duckweed microbiomes potentially influence plant traits on growth, nutrition, and stress tolerance*”). While earlier taxonomic profiling using partial 16S rDNA sequencing provided initial insights into duckweed microbiome composition (e.g., [24,25,26]), metagenomic sequencing now offers deeper understanding of their functional roles. Specifically, her research group successfully reconstructed several metagenome-assembled genomes (MAGs) from the *W. globosa* microbiome, uncovering potential metabolic pathways—such as synthesis of trehalose, betain, and gamma-aminobutyrate—that could significantly influence host plant characteristics.

The research groups of Eric Lam, Klaus-J. Appenroth, and Osnat Gillor have recently focused on cobalamin (vitamin B12) production by duckweed-associated bacteria, exploring its potential applications in human nutrition [27,28]. Rhishika Dutta (Ben-Gurion University, Israel), from Gillor’s lab, presented insights into synergistic interactions within the duckweed endophytic microbiome related to cobalamin synthesis (“*Microbial cooperation in duckweed microbiomes: unraveling mechanisms of cobalamin biosynthesis*”). Her findings revealed that cobalamin biosynthetic genes are distributed across multiple bacteria within the *W. globosa* endophytic community. Intriguingly, while none of the isolates from the endophytic community produced cobalamin when cultured individually, successful production was observed when these strains were cultured in pairs or triads. These results suggest that duckweed endophytic bacteria, which often possess incomplete sets of cobalamin biosynthetic genes, rely on synergistic metabolic relays among co-existing species to produce cobalamin. Huyền Thị Thanh Phạm from Masaaki Morikawa’s laboratory, Hokkaido University, Japan, reported a novel strategy to enhance duckweed production in the environment by leveraging microalgal growth-inhibiting bacteria (MGIB) (“*Reconstruction of a functional duckweed holobiont to reduce nutrient competition with microalgae for high-yield biomass production*”). Since microalgal overgrowth is a common challenge that reduces duckweed yield, her study aimed to design a duckweed-microbe holobiont capable of antagonizing harmful microalgae. Screening experiments identified two MGIB strains that effectively inhibit the growth of model microalga, *Coelastella* sp. Furthermore, co-inoculating these MGIB strains with plant growth-promoting bacteria (PGPB) not only mitigated the adverse effects of microalgae but also stimulated duckweed growth, resulting in an 84.5% increase in *L. aequinoctialis* yield [29]. These findings suggest that leveraging microbial functions, combined with strategies such as duckweed coverage control, offers an effective means to manage microalgal overgrowth and enhance duckweed production.

## 6. Applications

During this 7th ICDRA, there was a strong focus on the applications of duckweeds especially in the fields of nutrition and wastewater treatment. Use of duckweed as human food was well presented by the variety of dishes offered during the conference’s welcome dinner included cakes and ice creams made with *Wolffia*. The presentations in this section were opened by the invited speaker Ingrid M. van der Meer, Wageningen University & Research, the Netherlands, on the topic, “*Lemna: from novel food application to the market*”. Duckweed, not having been traditionally eaten in Europe, is considered as a “novel food”. The several steps involved in the process of a novel food application for duckweed as a vegetable in Europe were discussed in her talk. Also, the real-time limitations in the process were well discussed. These aspects are especially important for translating the lab-scale knowledge to the commercial market.

The oral presentations in this section mostly focused on the use of duckweed for treatment of wastewater from different sources. Marie Lambert, Inagro vzw, Belgium, in her presentation on the topic “*Monitoring a duckweed-based remediation system for nutrient recovery from pig manure into feed*”, discussed the efficiency of a constructed wetland at a pig farm manure processing plant in allowing *L. minor* to remove nutrients from the waste and produce a protein-rich biomass which was comparable or higher than other similar systems. The results were also interesting from the point of view that the testing was carried out for a full growing season in a temperate maritime climate. The use of duckweed for treatment of dairy wastewater was investigated by Kari Lagan, The Pennsylvania State University, USA, as presented in her talk entitled “*Leveraging acidification pretreatment of dairy digestate to increase duckweed protein yields while decreasing ammonia volatilization and water use*”. One of the major limitations of the efficient use of duckweed for dairy wastewater treatment is the presence of high levels of ammonia. Acidification pretreatment to convert ammonia to ammonium which can be used by the plants was shown as a solution to this problem. “*Methane production potential from co-digestion of Spirodela polyrhiza and tapioca starch wastewater*” was presented by Thanapat Thepubon, Khon Kaen University, Thailand. It was shown that the addition of duckweed to the tapioca starch wastewater increased methane production under anaerobic conditions. This has applications in the bioenergy sector. In the last presentation of the section, Werawich Pattarayingkul, Kasetsart University, Thailand, presented her results on the topic: “*The impact of drum drying on the amino acid profile and protein digestibility of Wolffia globosa*”. This presentation addressed a post-harvest processing problem for the long-term use of duckweed as a protein source. It was shown that the process of drum drying of the duckweed did not have any significant negative effect on the protein quality and digestibility.

Furthermore, a panel discussion on the applications of duckweeds was organized, emphasizing the topic “*Experience Sharing: Challenges in Large-Scale Duckweed Cultivation, Product Innovation, and Commercialization”*. Metha Meetam, Advanced Greenfarm, Thailand; Tsipi Shoham, GreenOnyx, Israel; Kayleigh Dugas, Fyto, USA; and Sven Kaufmann, Sustainable Planet, UK, shared their valuable insights and experiences. The challenges faced in indoor and outdoor large-scale cultivation of duckweeds were discussed.

## 7. Future of Duckweeds: Nine “Famous” Problems in Duckweed Research and Applications

During the congress of mathematicians in 1900 in Paris, France, David Hilbert (1862–1943) announced a famous list of 23 mathematical problems which mathematicians should pay more attention to [30], now known as “Hilbert’s problems”. More than 120 years later, most of them are solved. In his introductory lecture, Klaus- J. Appenroth mentioned nine “famous” problems in the field of duckweeds where more effort in research and applications is needed. We suppose that these problems will be solved in a shorter time than Hilbert’s ones.

*1. Industrial production of biomass*

Large scale production of duckweed biomass in aquaculture is a precondition for any industrial application. As this problem is not yet satisfactorily solved, it currently presents the basic problem. The methods depend on the intended use of biomass. Future will show whether outdoor cultivation or indoor cultivation under unsterile or sterile conditions are more effective. “Sustainable Planet” represents one successful example for outdoor cultivation in Mozambique [31], and GreenOnyx, Israel, is another example for sterile indoor cultivation.

*2. Large scale, industrial production of protein OR starch*

The use of duckweed biomass for feed and food requires high protein-containing biomass, whereas for the production of energy, a high content of starch is useful. High protein content is obtained after cultivation under optimal growth conditions [32], whereas high starch content is obtained under certain stress conditions [33,34] and under high CO_2_ concentrations [35]. The results of lab-scale experiments need to be translated to commercial level.

*3. Taxonomy of duckweeds*

Reliable identification of duckweed species and clones of the same species is indispensable to produce reproducible results so as to follow the rules of good scientific practice. In many cases, a combination of molecular methods (e.g., plastidic barcoding and tubulin-based polymorphism) is necessary, as already shown by some groups of species of the genus *Lemna* [36]. In the near future, *Wolffiella* and *Wolffia* genera must also be investigated with these methods.

*4. Further genome sequencing*

At present, genomes of few species representing all five genera of Lemnaceae are sequenced (summary in [2]). For proteomic, metabolomic, and other investigations, the genome data of all 35 species (and hybrids) are required. Moreover, because of the intraspecific diversity of physiological properties, several clones of the same species should be sequenced [37].

*5. Genetic transformation*

Genetic transformations are required to employ the full capacity of these plants for research and applications. Several reports were published over the years, e.g., by Wei et al. [38]. For using the *Agrobacterium* system on large scale, the efficiency must be improved. Employing the CRISPR/Cas9 system, transformation of *L. aequinoctialis* was reported [39]. More standardizations in this direction are required.

*6. Single cell transcriptomics—for duckweed development*

One of the first reports about single cell transcriptomics was by Denyer et al. [40] using *W. australiana*, which was also reported during this conference. Single cell transcriptomics has a huge potential to investigate developmental processes in duckweed, e.g., turion formation [41]—although this is challenging.

*7. Interaction with microorganisms*

The interaction of duckweed with microorganisms, especially bacteria, has been known for a long time (for a review cf. [42]), but the molecular mechanisms need to be better understood. Some results are already available for the acceleration of growth [43,44], and, very recently, for the production of vitamin B12 by duckweed-associated bacteria in a large number of duckweed species [28].

*8. Wastewater phytoremediation*

The group of Marcel A.K. Jansen at the University College Cork, Ireland, is not only testing the cleaning of wastewater from very different sources, they are also going ahead with upscaling from laboratory experiments to pilot plants under the given climatic conditions [45]. Another problem to be solved consists of the fact that the resulting biomass must be used considering the influence of the specific wastewater contaminations.

*9. Duckweeds for human nutrition*

The use of duckweed for human nutrition is promising because of the higher prices compared to animal feed in the commercial market. However, the requirement for cultivation conditions is also higher. Clones with the highest protein contents have to be selected. Moreover, the concentration of possible anti-nutritives must be measured; related experiments are running in the groups of K-J. Appenroth at the University of Jena, Germany, and K. Sowjanya Sree, Banaras Hindu University, India. Thereafter, problems that are hardly considered by duckweed producers, e.g., post-harvest processes during industrialization and commercialization, have to be tackled [46].

## Data Availability

No new data were created or analyzed in this study.
